# Metal–Organic Framework for the Immobilization of Oxidoreductase Enzymes: Scopes and Perspectives

**DOI:** 10.3390/ma16196572

**Published:** 2023-10-06

**Authors:** Pengyan Yang, Wenhui Yang, Haiyang Zhang, Rui Zhao

**Affiliations:** 1 School of Light Industry, Beijing Technology and Business University (BTBU), Beijing 100048, China; 2 Department of Biological Science and Engineering, School of Chemistry and Biological Engineering, University of Science and Technology Beijing, Beijing 100083, China

**Keywords:** oxidoreductase, metal–organic framework, immobilization, multi-enzyme cascade

## Abstract

Oxidoreductases are a wide class of enzymes that can catalyze biological oxidation and reduction reactions. Nowadays, oxidoreductases play a vital part in most bioenergetic metabolic pathways, which have important applications in biodegradation, bioremediation, environmental applications, as well as biosensors. However, free oxidoreductases are not stable and hard to be recycled. In addition, cofactors are needed in most oxidoreductases catalyze reactions, which are so expensive and unstable that it hinders their industrial applications. Enzyme immobilization is a feasible strategy that can overcome these problems. Recently, metal–organic frameworks (MOFs) have shown great potential as support materials for immobilizing enzymes due to their unique properties, such as high surface-area-to-volume ratio, chemical stability, functional designability, and tunable pore size. This review discussed the application of MOFs and their composites as immobilized carriers of oxidoreductase, as well as the application of MOFs as catalysts and immobilized carriers in redox reactions in the perspective of the function of MOFs materials. The paper also focuses on the potential of MOF carrier-based oxidoreductase immobilization for designing an enzyme cascade reaction system.

## 1. Introduction

Oxidoreductases, as a wide class of enzymes, play an important role in regulating redox reactions by catalyzing the transfer of electrons from one molecule to another. Recently, many primary oxidoreductases, such as glucose oxidase (GOx), laccase(Lac), peroxidase (POx), and catalase (CAT), are now being widely employed industrially in a variety of biotechnological processes. The area of application involves biocatalysis [1], biosensing [2,3,4], the degradation of pollutants [5,6,7], medical diagnostics [8], and others.

In terms of free oxidoreductases, there are some deficiencies that limit their industrial use. That is, (1) the redox reaction, as a fundamental and significant activity in the organism, relies on electron transfer. Oxidoreductases themselves cannot gain or lose electrons. Therefore, the majority of oxidoreductase-catalyzed reactions necessitate the presence of cofactors. Cofactors provide the electrons needed for the reaction. However, the coenzymes are relatively expensive and difficult to recycle in the reaction. It is economically constrained by free oxidoreductases in practical production. (2) Oxidoreductase has a large number of cofactor and substrate binding sites and is usually composed of several subunits. This precise structure easily results in enzyme inactivation during catalysis, which shows poor stability; (3) Free oxidoreductase is difficult to separate from the product in biocatalysis. 

In order to improve the above deficiencies, one of the most useful strategy is to bind or confine the enzyme in a specific space with a carrier material, which is called immobilized enzyme technology. This technology could improve the catalytic activity, storage stability, and thermal resistance of the enzyme. Additionally, immobilized enzymes could be easily separated from the reaction mixture without contaminating the product. Especially for oxidoreductases, enzyme immobilization technology is not only helpful for simple enzyme catalytic reactions, but also plays a significant role in biological cascade catalytic systems. Since oxidoreductase reactions require the participation of coenzyme, the coenzyme regeneration enzyme-based enzyme cascade reaction system can significantly increase the catalytic efficiency, lowering the cost of coenzymes. In addition, oxidoreductase could be combined with the other enzymes to catalyze synthesis of complex/advanced products as well. Immobilized enzyme technology not only improves the operating conditions of each step, but also lowers mass transfer resistance [9,10]. Therefore, immobilized enzyme technology has significant practical implications for oxidoreductases.

For enzyme immobilization, the structure of carrier materials could directly impact the performance of the biocatalytic system [11]. In recent decades, many kinds of organic, inorganic, and hybrid/composite materials with porous structures have been constantly emerging [12], including sol-gel [13], mesoporous silica [14], polymeric materials [15,16], and metal- and carbon-based materials [17,18]. However, there are still problems, like low protein loading efficiency, low stability, and enzyme leaching, that should be solved. Researchers are making great efforts to develop novel carriers with adaptable surface structures and variable physical characteristics to increase the stability and catalytic efficiency of immobilized enzymes.

Metal–organic frameworks (MOFs) are extremely crystalline and porous materials composed of metal ions or metal clusters and organic molecules (ligands) by coordinated interaction to form one-, two-, or three-dimensional structures. In recent years, the utilization of MOFs in the field of enzyme immobilization has gradually increased. Compared to traditional porous materials, MOFs have some excellent advantages, including an ultra-high surface area and tunable ultra-high porosity, which increase the loading capacity of the enzyme. Additionally, the unique pore channel of MOFs could maintain the immobilized enzyme’s conformational structure, making the enzyme incredibly stable. The functional designability and good thermal stability of MOFs themselves also offer great potential for the application of enzyme@MOFs in different fields.

In this paper, the application of MOFs and their composites in oxidoreductase catalysis is reviewed from the perspective of the role played by MOFs. The paper pays more attention to the potential of MOFs in the carrier-based immobilization of oxidoreductase and as catalysts in the design of multistage enzymatic reactions.

## 2. MOFs as the Oxidoreductase Carriers

**Figure 1:**
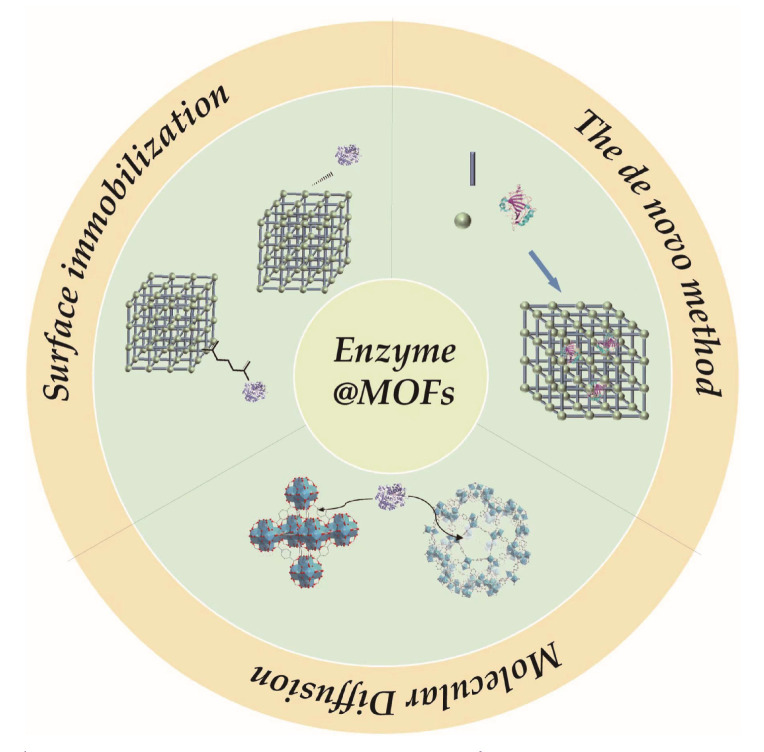
Synthesis of enzyme@MOFs.

The application of free oxidoreductase is usually constrained by its weak stability and easy inactivation. MOFs, which have well-defined pores, great chemical-thermal stability, and extremely high specific surface areas, are a class of ideal carriers for oxidoreductase immobilization. Recently, many kinds of oxidoreductases, including Lac, CAT, POx, horseradish peroxidase (HRP), etc., have been successfully immobilized on the MOFs, whose catalytic activity and stability are increased. According to the method of immobilization, there are two strategies employed to immobilize oxidoreductase, which are the de novo method and post-synthetic modification (surface immobilization and molecular diffusion), as illustrated in Figure 1. 

For the de novo method, the enzyme molecule is initially added into the MOF precursor solution, which serves as the core. MOF crystals subsequentially in situ grow around the enzyme [19]. This method has no requirement on the size of MOFs and can reduce the leaching rate of enzymes. For the post-synthetic modification, MOF carriers are constructed without the presence of enzyme first, and the enzymes are then introduced onto or within the MOF materials. The post-synthesis modification method could be further divided into two pathways: surface immobilization, and molecular diffusion. Surface immobilization is usually achieved by physical adsorption or covalent attachment. In terms of physical adsorption, enzymes are placed on the surface of MOFs through hydrogen bonding, van der Waals forces, and electrostatic interactions. Factors such as dipole moment, magnetic susceptibility, polarizability, and electric charge, affect the surface properties of the two materials, which in turn determine the strength of adsorption between enzymes and MOFs. On the other hand, tighter attachment of the enzyme onto MOFs can be achieved using covalent bonds, which are usually formed by an amide reaction between the free amino group on the surface of the enzyme or MOFs and the carboxyl group on the surface of the enzyme or MOFs [20,21,22]. Similarly, enzyme immobilization using the molecular diffusion approach is achieved by physical adsorption or covalent attachment. However, the -place of immobilization is the pores of mesoporous MOFs instead of the surface of the MOFs. Enzyme firstly diffuse into the channels of MOF, and then specifically interact with the functional groups of MOFs. Compared to surface immobilization, molecular diffusion prevents the aggregation or folding of enzymes. The stability of enzyme could be improved via the protection of the pore channel [23,24,25].

The immobilized oxidoreductase prepared by the above method could exhibit higher stability. On the one hand, MOFs could serve as a protective shield against harsh conditions due to the structurally balanced and controllable pore morphology of MOFs [26]. Accordingly, the thermal stability, OH stability, medium stability, and the storage stability of enzyme@MOFs could be improved. On the other hand, the presence of the required hydrophilic/hydrophobic groups in MOFs and the strong electrostatic interactions between MOFs and enzymes was helpful to increase the binding efficiency of the enzyme to the substrate, water stability, and reusability [22,27].

### 2.1. The De Novo Method

The de novo method, which is called the one-pot approach, in situ encapsulation, co-precipitation, biomimetic mineralization, etc., involves the production of hierarchically structured materials through the self-assembly of organic and inorganic building blocks. This concept is derived from in situ biomimetic mineralization. It has been demonstrated that biomolecules, such as enzymes, could serve as seeds for the mineralization process. MOF building blocks could aggregate around these biomolecules through intermolecular hydrogen bonds, electrostatic interactions, and hydrophobic interactions [28].

The catalytic activity of the immobilized oxidoreductase obtained using this method could be greatly enhanced. Especially for Lac, this technology showed great application potential in the field of pollutant degradation. Wei et al. reported for the first time the complete removal of eliminated phenol, p-cresol, and p-chlorophenol by immobilizing tyrosinase on Zeolite imidazole framework-8 (ZIF), which showed high efficiency in the degradation of phenolic pollutants [29]. Jiang et al. prepared Lac at Cu (PABA) using an efficient one-pot approach. A total of 92% of the dye was removed within 6 h, and 70% of the dye could be removed after 10 cycles [30]. Molina et al. adopted the in situ encapsulation approach to improve the loading capacity of Lac in NH_2_-MIL-53 (Al) [31]. The resulting biocatalysts had a 100 mg/g enzyme loading without enzyme leaching when removing biphenol from wastewater. Besides Lac, in situ encapsulation was also used to immobilize HRP on the ZIF-8 molecular sieve membranes. The efficiency of bisphenol removal was up to 98% [32]. Wu et al. prepared HRP/ZIF-8 nanocrystals with a homogeneous particle size and morphology using the co-precipitation method in reverse micelles. Compared to free HRP, the activity increased 2–5 times [33].

The stability and reusability of the immobilized oxidoreductase obtained by this method were also improved. Tocco et al. used one-pot synthesis to immobilize Lac in Fe-BTC and ZIF-zni, respectively. Lac@Fe-BTC could be stored at 4 °C for five days without the enzyme activity declining. Lac@ZIF-zni could keep 50% of its initial enzyme activity after thirty days [34]. Though the de novo method of encapsulating the enzyme in MOFs could increase its activity and stability, it might result in a slower rate of enzyme reaction due to less opportunity for the substrate to bind to the enzyme’s active sites. [35].

### 2.2. Surface Immobilization

Oxidoreductase could be immobilized on the surface of MOFs by physical adsorption or covalent attachment, which had the broadest variety of applications and favorable mass transfer abilities. By using covalent and adsorption techniques, CAT was immobilized on MIL-125-NH_2_. The CAT@MIL-125-NH_2_ was able to maintain 50% of the initial activity and 62% of the residual activity after 14 days of storage at room temperature, demonstrating good reusability [36]. Pang et al. immobilized Lac on a novel bimodal microporous Zr-metal–organic framework (Zr-MOF, MMU) through physical adsorption. This unique structure could prevent Lac from being overcrowded, which avoided the inactivation of enzyme and clogging of MOF pores. Under harsh conditions, the immobilized Lac exhibits superior stability and repeatability [37]. In order to physisorb HRP into mesoporous pores and immobilize it there, Gao et al. also produced a hierarchical porous Zr-MOF. The resulting cementation capacity of immobilized HRP was up to 61.6 mg/g [38].

In addition, in order to further increase the enzyme loading capacity and activity, a cross-linking strategy could also be employed to further immobilize the enzyme molecules on the MOF carrier. Glutaraldehyde was a typical cross-linker used in enzyme immobilization. Li et al. used it to further fix Lac on the surface of Cu-MOF. The loading capacity was as high as 128.48 mg/g, and the activity was 2.57 times higher than that of the free enzyme activity [39]. Similarly, after enzyme crosslinking, immobilized Lac on aminated ZIF-8 could be used as green nano-biocatalysts for the treatment of industrial wastewater [40]. However, covalent attachment, including covalent-crosslinking, tended to lead to conformational changes in the enzyme-reducing enzyme activity.

### 2.3. Molecular Diffusion

Surface immobilization might lead to the waste of the MOFs’ pores, enzyme aggregation, mutual attraction between enzymes, and exposure of the enzyme to harsh surroundings. In order to overcome the above problem and make full use of the pore channel and functionality of MOFs, building MOFs with a macro-porous structure was an effective strategy. There were two approaches used to introduce mesopores in MOFs, including the introduction of defects and nucleation kinetics. The introduction of defects was accomplished mostly by replacing a portion of the metal or ligand in the MOF. The nucleation kinetics strategy enabled the formation of tunable mesopores by changing the synthesis conditions.

By using the nucleation kinetics strategy, a water-stabilized [PCN-333(Fe)] with an extra-large cavity and extremely high porosity was synthesized. HRP could enter the interior of the carrier, which was applied as a biosensor with high sensitivity (a linear range of 0.5 mu M to 1.5 mM and a low detection limit of 0.09 mM (S/N = 3)) [41]. Gkaniatsou et al. immobilized microperoxidase8 (MP8) and microperoxidase11 (MP11) into the mesoporous MOFs, which improved the activity and stability in the oxidation of dyes and phenol derivatives [42]. Zhong et al. reported that the loading capacity of Lac in mesoporous Cu-MOFs could reach 502 mg/g, and activity recovery rate was up to 95.2% [43].

The mixed ligand strategy required large organic linkers that may be expensive and difficult to synthesize MOFs. Up to now, there has been scarce study on the immobilization of oxidoreductase using the mixed ligand strategy to form macro-porous MOFs. Liu et al. constructed a leak-proof Zr-based MOF carrier, UiO-66-NH_2_ (30). The confocal laser scanning microscope (CLSM) results indicated immobilized Lac not only on the surface but also in the interior. The enzyme loading capacity was 275.96 mg/g with high enzyme activity recovery [44].

Compared to the other methods, molecular diffusion immobilized the enzyme inside mesoporous MOFs, which was both easy to manipulate and improved enzyme stability. However, the challenge of this method was the construction of the MOFs with suitable macropores.

## 3. Composite MOFs as the Oxidoreductase Carriers

The fabrication of enzyme@MOF composites with polymers, natural chemicals, etc. could increase the selectivity and binding affinity of the enzyme to the substrate. In addition, enzyme@MOF composites taking advantage of the other material could enhance the catalytic activity or the electron transfer rate of the redox reaction, and prevent enzyme leaking. Therefore, MOF-based composites could be more widely used in enzyme immobilization. In recent studies, the enzyme@MOF composites were frequently combined with other inorganic or organic materials and applied in biosensors, biocatalytic processes, and diagnostic diseases [45]. 

### 3.1. The Application of Oxidoreductase@MOF Composites in Biosensors 

Biosensors have been currently receiving a lot of attention in the field of biometrics, chronic disease management and clinical diagnosis, etc. When preparing oxidoreductase-based biosensors, enzymes were usually immobilized on functionalized carriers. MOFs with high specific surface area and framework integrity could serve as carriers for oxidoreductase immobilization to improve catalytic performance and enzyme stabilization. Additionally, nanometal particles, hydrogels, biofactors, and other materials were commonly employed to make oxidoreductase@MOFs meeting the demands of biosensor application. Among a range of novel oxidoreductase biosensors, the glucose biosensor was one of the most widely used. 

#### 3.1.1. Glucose Sensors

**Figure 2:**
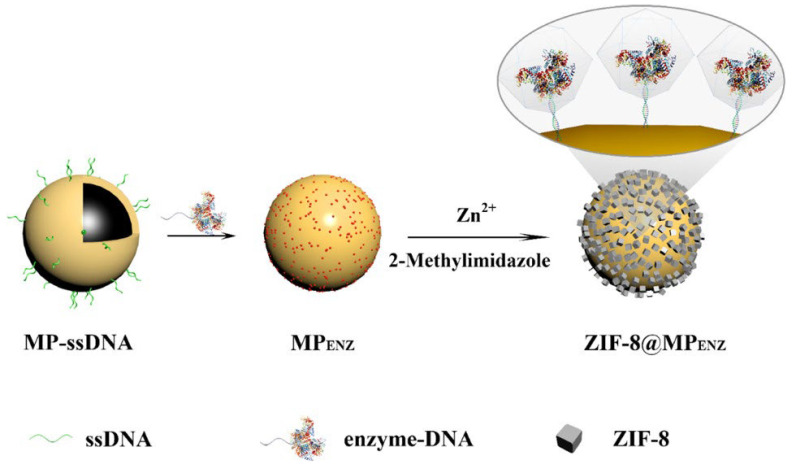
Schematic Synthesis of ZIF-8@MP_ENZ_. Reprinted with permission from Ref. [51].

Chen et al. synthesized copper-based MOFs with multi-walled carbon nanotube (HKUST-1-MWCNTs) composites by a one-step hydrothermal method. The obtained material was used to immobilize GOx for the preparation of biosensors. The biosensor achieved a sensitive amperometric detection of glucose at 0.7 V [46]. Moreover, some novel glucose biosensors were fabricated by immobilizing GOx on ruthenium-based conjugated polymer (CP) and MOF nanocomposites, and platinum nanoparticles (Pt NPs) decorated reduced graphene oxide (rGO)/Zn-MOF-74 hybrid nanomaterials [47,48]. Li et al. encapsulated enzymes into the MOFs and then robustly anchored it to the cellulose acetate (CA) nanofiber membrane to prepare a highly flexible electrode. Over 15 h of continuous long-term monitoring, the sensor showed excellent stability [49]. Zhong et al. reported a novel biosensor by co-encapsulating GOx and POx into a defective MOF that was alginate gelation-coated. This hybrid biosensor could sensitively and selectively detect glucose with a linear range of 0.05 to 4 mM [50]. Zhou et al. in situ encapsulated DNA–enzyme composites in ZIF-8 and subsequently immobilized them on the surface of magnetic particles (MPs), as shown in Figure 2. This method could be used to stabilize various proteins. ZIF-8@MPGOx-HRP had high selectivity and a wide linear range (25–500 mu M) for glucose detection [51]. Moreover, GOx could also be co-immobilized with platinum nanoparticles or TiO_2_ nanoparticles on Zr-MOF and ZIF-8 to prepare glucose sensors [52,53]. Zhu et al. developed label-free optical fiber biosensors with high stability by encapsulating GOx into ZIF-8 and combining it with long-period grating (LPG). It showed linear response with glucose solutions range from 1 mM to 8 mM with a sensitivity of about 0.5 nm/mM [54].

#### 3.1.2. Other Sensors

Cunha-Silva prepared a screen-printed biosensor by cross-linking lactate oxidase at Cu-MOF on a chitosan-covered platinum-modified surface. RSD values of the biosensor was less than 7% in both the catalytic and inhibitory regions, which showed good accuracy [55]. Li et al. prepared a sensitive sandwich-type electrochemical immunosensor, Ce-MOF@ hyaluronic acid (HA)/Ag-HRP, to catalyze H_2_O_2_ and double-amplify the current signal [56]. Huang et al. prepared the amorphous MOFs coated with PtCu hydrogels as multifunctional carriers to encapsulate HRP. The PtCu@HRP@ZIF-8-based biosensor was applied for the sensitive sensing of organophosphorus pesticides (OPs). The proposed biosensor exhibited a favorable linear relationship with a concentration of paraoxon-ethyl from 6 ng/mL to 800 ng/mL and a low detection limit of 1.8 ng/mL. This biosensor showed great potential for practical applications [57].

### 3.2. Biocatalysis

Some functional materials were also combined with the MOFs before or after enzyme immobilization, which played a part in reducing the leakage of the enzyme. This strategy could enhance the loading capacity, catalytic activity, durability, and reusability of the enzyme.

#### 3.2.1. Organic Materials

Gao et al. prepared MHCo_3_O_4_ and Co/C and MHAl_2_O_3_ MOFs using the self-sacrificial template strategy. The obtained materials were then wrapped with polydopamine (PDA) and entrapped with HRP. Compared with to the free enzyme in bulk buffer, the functionalized “PDA” bionic membranes on the surface of the carriers could enhance substrate specificity and binding affinity. When using this immobilized HRP, 2 mmol/L 2,4-dichlorophenol was completely degraded in 15 min [58], and the degradation rate reached 82.7% in 30 min [59]. Li et al. synthesized a graphene aerogel–zirconium MOF (GA-Zr-MOF) to immobilize Lac. The removal rate of hydroquinone was 79%, and the removal efficiency remained around 70% after five cycles [60].

#### 3.2.2. Inorganic Materials

**Figure 3:**
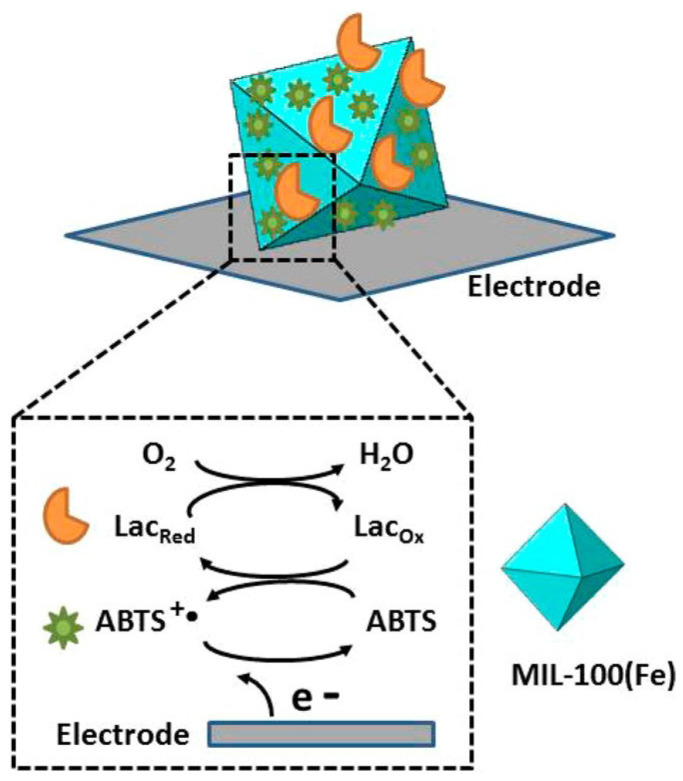
Schematic presentation showing pathways of electron transfer from the electrode to molecular oxygen. Reprinted with permission from Ref. [62].

In order to improve the reusability of oxidoreductase, Lou et al. developed a co-immobilization method for Lac and 2,2′-Azinobis(3-ethylbenzothiazoline-6-sulfonic Acid Ammonium Salt) (ABTS) on Poly(ethylene terephthalate) (PET)/UiO-66(Zr)-NH_2_. The obtained immobilized enzyme showed better stability than free Lac under acidic conditions, which could be applied to the practical treatment of dyes in wastewater [61]. As depicted in Figure 3, Patra et al. also co-immobilized MIL-100 (Fe), Lac, and ABTS, which showed good stability over 3 weeks [62].

### 3.3. Biomedical

In the biomedical applications, MOFs were typically combined with gel-like or bio-nano mediators in order to improve the biocompatibility, biodegradability, and non-toxicity of materials. Up to now, researchers have developed many kinds of MOF composites to immobilize GOx and HRP, and applied them in the treatment of cancer and bacterial wound infections, such as chemodynamic therapy, enzyme-activated prodrug therapy, synergistic starvation therapy, as well as antibacterial and anti-inflammatory uses.

#### 3.3.1. Cancer Treatment

Chemodynamic therapy (CDT) was an emerging cancer treatment that relied on Fenton or Fenton-like reactions to kill cancer cells. Due to good biocompatibility and biodegradability, hyaluronic acid was commonly used as a targeting component for enhancing cancer therapy performance and inhibiting metastasis. Researchers used hyaluronic acid to combine with a variety of drug-loaded nanoparticles or enzyme composites, such as Cur@NH_2_-MIL [63] and Cu-MOF@GOx [64] to enhance the efficacy of CDT and inhibit metastasis. Pan et al. reported a copper (II)-based metal–organic nanoframework (MOF) loading disulfiram prodrug (DQ) and GOx conjugates for enhancing chemokinetic treatment efficacy [65].

**Figure 4:**
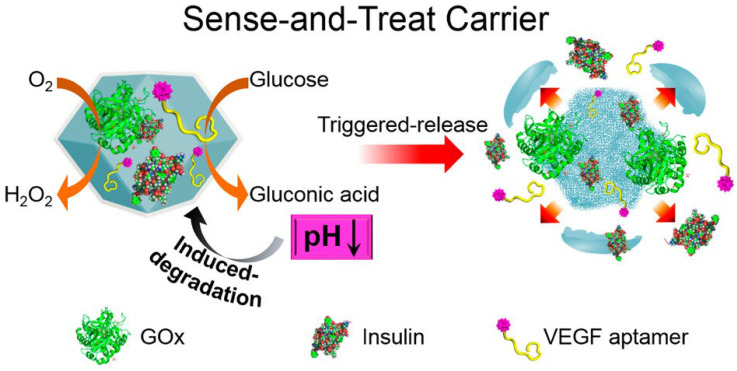
Schematic synthesis of the insulin/GOx-loaded ZIF-8 NMOFs and the pH-induced degradation of the NMOFs through the GOx-catalyzed oxidation of glucose. Reprinted with permission from Ref. [66].

Chen et al. co-immobilized insulin and GOx in ZIF to develop sensing and therapeutic systems (Figure 4) [66]. GOx and 1-methyltryptophan (indoleamine 2,3-dioxygenase (IDO) inhibitor) were loaded on a MOF nanoreactor. It could amplify release of tumor starvation/oxidative immunotherapy [67]. Some researchers fabricated a versatile bioreactor by embedding GOx and doxorubicin (DOX) in MOFs. This multifunctional bioreactor could control drug delivery and was employed for synergistic starvation therapy [68,69].

Wang et al. developed an effective cancer treatment strategy called enzyme-activated prodrug therapy by encapsulating HRP and indole-3-acetic acid (IAA) prodrugs in layered porous MOFs [70]. Additionally, ZIF-8 nanoplatforms were used to co-deliver alpha-cyano-4-hydroxycinnamate (CHC) and GOx for cancer therapy [71].

#### 3.3.2. Antimicrobial and Anti-Inflammatory

Antibiotic resistance by bacteria and persistent inflammation were key challenges in treating bacterially infected wounds. Therefore, the development of multifunctional wound dressings with efficient antimicrobial properties and inflammation modulation was imminent. Oxidoreductase immobilized in catalytically active MOFs and hydrogel was a feasible method. This prepared hydrogel dressing could reduce the risk of enzyme leakage, which also had flexibility, obvious water retention ability, good biocompatibility, anti-inflammatory effects, and antibacterial effects. It exhibited potential applications in biomaterials effective against bacterial infection.

Tian et al. successfully constructed a hydrogel wound dressing consisting of a bimetallic MOF and GOx, termed MOF (Fe-Cu)/GOx-polyacrylamide (PAM) gel, to accelerate wound healing [72]. Similarly, GOx@Fe-MOF was anchored on electrospun PCL/gelatin/glucose composite fibrous mesh through amide-bond-coupling formation. This wound dressing exhibited good bactericidal ability [73]. Zhang et al. encapsulated a GOx@ MOF-based nano-catalyst in bacterial cellulose (BC)-enhanced hydrogels to improve the ability of synergetic antibacterial defense and hemostasis [74]. Zhang et al. immobilized GOx/carbon dots on copper MOF nanofibers (GOx/CDs @MOF NFs) to develop a novel multifunctional wound dressing. This dressing could inhibit bacterial infection while visually monitoring the wound pH [75].

## 4. The Application of MOFs in Oxidoreductase Cascade Reactions

With the rapid development of biosynthesis technology, single enzyme catalysis could no longer meet the practical needs, and products of multi-enzyme cascades were widely used in biosensors, biocatalysis, and other industry. Therefore, developing immobilized multi-enzyme technology for cascade reactions to produce advanced products in the focus of future research was of great importance.

The multi-enzyme carrier should be biocompatible to protect them from harsh external environments, helpful for the transport of substrates and products, and stable to make the enzymes reusable. MOF materials have received increasing research attention due to their rich structural designability, versatility, large specific area, and porous nature [76]. In addition, the abundant metal sites in MOFs were expected to be activators of enzymes, consequently improving their biocatalytic performance [77]. Research on the construction of oxidoreductase cascade systems immobilized on MOF materials includes dual-enzyme co-immobilization, enzyme and biofactor co-immobilization, and single oxidoreductase immobilization by catalytically active MOFs.

### 4.1. MOFs as the Oxidoreductases Carriers for Catalyze Cascade Reactions

The immobilization of dual oxidoreductases on MOFs materials is the most common in enzyme cascade reactions. Moreover, there are still a small proportion of enzyme cascade reactions that are carried out by co-immobilizing oxidoreductases and pseudo-peroxidases. The pseudo-peroxidases are not enzymes in the traditional sense but could exhibit peroxidase-like behaviors when exposed to certain environmental factors.

#### 4.1.1. MOFs as the Dual Oxidoreductases Carrier

For dual oxidoreductase immobilization, the MOFs could provide a comfortable and relatively closed micro-space for each kind of enzyme to maintain high catalytic activity, due to the sufficient pore structure of MOFs. Researchers have encapsulated model oxidoreductases (GOx and HRP) in hierarchical porous MOFs [78,79,80,81,82,83], such as HKUST-1 and ZIF-8. This immobilized strategy not only significantly increased the overall biocatalytic efficiency but also maintained the catalytic activity over multiple catalytic cycles. Ahmad et al. immobilized GOx and HRP on two MOFs, UiO-66 and UiO-66-NH_2_, respectively. The enzyme activities of HRP/GO(x)@UiO-66-NH_2_ composites and HRP/GO(x)@UiO-66 were 189 U/mg and 143 U/mg, respectively. They also immobilized GOx and chloroperoxidase (CPO) on UiO-66 and UiO-66-NH_2_ to develop an enzyme system, which showed good catalytic activity [84,85]. Gao et al. prepared CPO/HRP at H-MOF (Zr), and the immobilized enzyme exhibited good stability at elevated temperatures. The activity at 70 °C for 1 h of incubation was increased by 582.2% compared to free enzymes. After 12 cycles, 70.7% of activity remained, which demonstrated that this composite has application potential in wastewater treatment [86].

**Figure 5:**
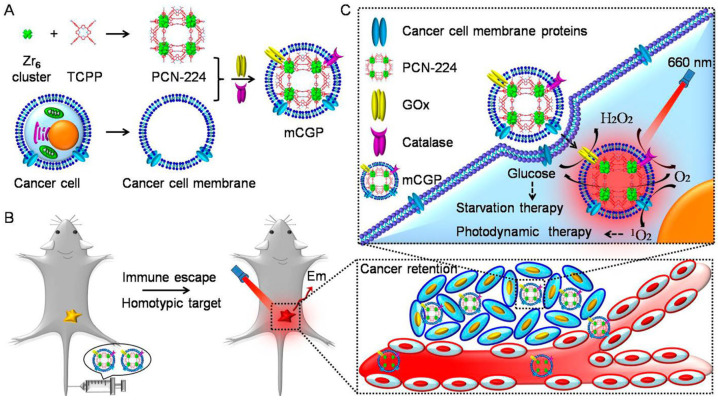
Schematic illustration of the cancer cell membrane camouflaged cascade bioreactor for cancer targeting starvation therapy and PDT. (**A**), The preparation processes of mCGP (**B**), The immune escape and homotypic targeting abilities of mCGP endowing cancer accumulation and retention behaviors after intravenous injection (**C**), The cascade reactions would amplify the synergistic effects of mCGP to cut off the cancer cell glucose supply for starvation therapy and promote O_2_ generation for PDT under light irradiation. Reprinted with permission from Ref. [87].

The immobilization of dual enzymes using MOFs was also widely used in the field of biosensors. By embedding GOx and CAT into PCN-224, a cancer-targeting cascade bioreactor was constructed for synergistic starvation and photodynamic therapy (PDT),which is shown in Figure 5 [87]. Liu et al. proposed a one-step strategy to construct a novel cascade bioreactor for the chiral sensing of multiple AA enantiomers by coupling L-amino acid oxidase (LAAO) and HRP into ZIF-8 (LAAO/HRP@ZIF-8) [88]. Liu et al. reported a facile and universal strategy for immobilizing GOx and uricase on HP-DUT-5. The maximum adsorption capacity of GOx and uricase was 208 mg/g and 225 mg/g, respectively. The constructed multi-enzyme biosensor showed good sensitivity, selectivity, and recoverability [89]. Table 1 summarized the representative examples of multi-enzyme cascade reactions in MOFs.

#### 4.1.2. MOFs as the Pseudo-Peroxidases and Oxidoreductases Carriers

In multi-enzyme cascades, pseudo-peroxidases was also co-immobilized with the oxidoreductase and employed in biocatalysis, biosensors, and cancer therapeutics. The widely used pseudo-peroxidases included hemoglobin, myoglobin, cytochrome C/cardiolipin complex, and cytochrome.

Chen et al. prepared a porous MOF as a host matrix for the immobilization of both GOx and bovine hemoglobin (BHb) to develop a cascade catalytic system with high catalytic properties [91]. Gao et al. prepared porous NiO (MHNiO) with a hierarchical structure as the HRP and cytochrome C carrier. Over 83% of its original activity was retained after incubation at 70 °C for 1 h [92]. GOx and hemoglobin were also encapsulated in ZIF-8, which was used for highly efficient cancer treatment [93]. A ferric MOF was covalently coupled with Pt(IV) prodrug and GOx to obtain a nanozyme MOF-Pt(IV)@GOx for cascade reactions [94]. Zhang et al. fabricated a biomimetic nanoreactor for starvation-activated cancer therapy by encapsulating GOx and the prodrug tirapazamine (TPZ) in erythrocyte membrane-cloaked MOF nanoparticles (TGZ@eM) [95].

### 4.2. Catalytic Cascade Reaction of Bifunctional MOFs Materials

**Figure 6:**
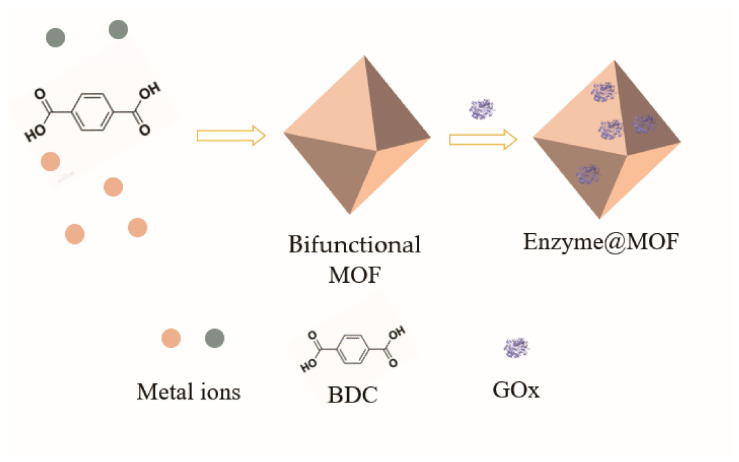
Synthesis of bifunctional MOF and schematic representation of the immobilized enzyme.

Conventional enzyme-immobilization carriers were chemically inert without catalytic activity [96]. By introducing metal ions into MOFs, the modified MOFs could promote the passage of hydrogen peroxide through the activation energy barrier and provide cofactors. Therefore, it could take the place of some oxidoreductases to catalyze some redox reactions. Here, MOFs could be used as carriers for oxidoreductase immobilization as well as another kind of catalyst to perform an enzymatic cascade reaction. Figure 6 illustrates the synthesis of a bifunctional MOF and the process of immobilized enzyme.

#### 4.2.1. Cu-Based MOFs

Cu-based MOFs were usually prepared by in situ doping during the crystallization or post-synthesis loading of copper ions. Herein, copper ions could effectively promote the generation of free radicals and electron transfer in the reaction process, which could induce peroxidase activity. Through the immobilization of other oxidoreductases on Cu-based MOFs, cascade reactors could be fabricated and applied in broader industrial areas. Yang and Huang et al. prepared Cu-MOF, which performs the dual functions of loading GOx and mimicking POx. The obtained enzymes were employed for the determination of glucose in human serum, which provided a more convenient and sensitive operating platform for bioanalysis [97,98]. Lin et al. immobilized a novel GOx in a Cu-hemin MOF with a ball-flower structure and used it as a bienzymatic catalyst for the detection of glucose [99]. Besides GOx, other oxidoreductases, such as Lac, were also immobilized on Cu_2_O@MOF via covalent linkage with synergistic catalytic activity [100]. Shahba et al. synthesized Cu-MOFs with peroxidase-like activity and used them as an effective immobilization matrix of choline oxidase (ChOx). ChOx@Cu-MOF had a great application perspective in bio-sensing and industrial catalysis [101]. Li et al. added Cu^2+^ when immobilizing GOx on ZIF. The co-delivery of Cu^2+^ and GOx could realize Cu^2+^/Cu^+^ cycling and the amplification of the Fenton reaction for antitumor experiments [102].

#### 4.2.2. Iron-Based MOFs

Various iron-based MOFs constructed by introducing iron ions had excellent peroxidase-like catalytic activity, which was similar to Cu-based MOFs [103]. Researchers also prepared various iron-based metal–organic skeleton materials to develop multi-enzyme cascade systems and applied them in detectors and diagnostics. Huang et al. constructed mesoporous MOFs with good POx mimetic bioactivity through an iron mineralization strategy. This material loaded GOx and could significantly enhance co-catalytic ability [104]. Xu et al. covalently immobilized GOx on Fe-MIL-88B-NH_2_, which exhibited significant POx activity, ultra-high stability, and high biocompatibility [105]. The MIL-88B(Fe)-NH_2_ material was also used as a nano-enzyme and carrier for glutamate oxidase (GLOX) immobilization [106]. Similarly, GOx was immobilized on MOF-545 (Fe) with POx activity to develop a simulated multi-enzyme system for tandem catalysis [107]. HP-PCN-222(Fe) with tunable graded porosity was synthesized, which acted as not only an efficient immobilization matrix for GOx, but also a POx mimic for glucose detection [108]. Zhao et al. prepared Fe(III)-BTC as a solid carrier for immobilized GOx and as a mimic of POx for the cascade colorimetric determination of glucose [109]. Yang et al. encapsulated GOx in Fe-doped imidazoline frameworks for the treatment of diabetic-infected wounds and other disorders [110]. Zhao et al. developed a MOF system (GOx@MOF@Fe^3+^) to enhance the therapeutic cascade response. With the help of electrolysis, this multi-enzyme catalyst was applied for starvation therapy and CDT [111].

#### 4.2.3. Bimetallic MOFs

**Figure 7:**
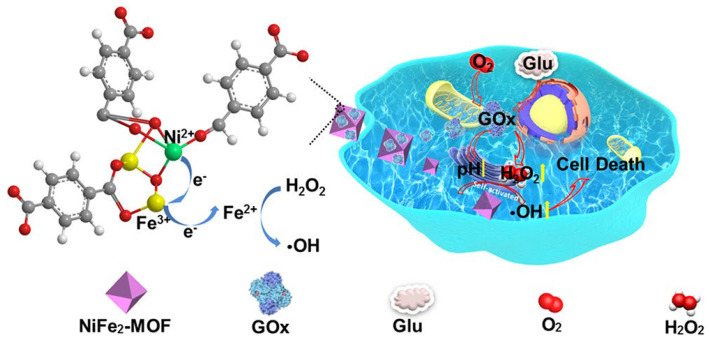
Schematic diagram of NiFe_2_ MOF/GOx as a self-activated cascade reagent for promoting·OH induction. Reprinted with permission from Ref. [116].

Bimetallic MOFs were constructed by introducing two different metal ions. By adding different proportions of two metal ions, the catalytic activity, catalytic rate, and stability of bimetallic MOFs could be further improved compared to single metal-based MOFs [112,113,114]. MOFs modified by two kinds of metal ions was also used for mimicking POx. Fang et al. synthesized nanoscale MOFs (Co-Fc NMOFs) with high Fenton activity. This material could further be coupled with GOx to develop a cascade enzyme/Fenton catalytic platform (Co-Fc@GOx) for enhancing oncology therapy [115]. Zhao et al. prepared bimetallic Ni and Fe-MOF to immobilize GOx as a self-activating cascade reagent. It could effectively induce cell death, as shown in Figure 7 [116]. Jiang et al. synthesized MIL-88(NH_2_)MOF-doped Co(II) and Fe(III) with HRP activity. After binding GOx, the cascade enzymatic reaction could be triggered for disease diagnosis and prognosis [117]. Liu et al. synthesized a bovine serum albumin-Pt nanoparticles@mesoporous MnCo_2_O_4_ (BSA-PtNP@MnCo_2_O_4_) MOF to immobilize GOx, which was used to build an enzyme cascade bio-platform. It achieved a superior detection of glutathione with a detection limit of 0.42 μM [118]. He et al. added Co^2+^ and La^3+^ to MOF-199 and then immobilized GOx, which has satisfactory anticancer effects [119].

#### 4.2.4. Other Metal MOFs

In addition to the copper- and iron-based MOFs, sub-100 nm peroxidase-mimetic zirconium porphyrin MOFs (Zr-PorMOFs) have also been successfully used to construct peroxidase-based tandem catalytic systems [120]. A new glucose sensor with highly sensitivity was constructed by immobilizing GOx on Co-MOF [121].

In summary, MOFs could be used as carriers to co-immobilize multiple oxidoreductases or oxidoreductases and pseudo-peroxidases. Moreover, it could also act as a catalyst combined with oxidoreductases to perform the cascade reaction. Due to the high catalytic activity, reusability, and stability of the enzyme, the cascade reactions of oxidoreductase have been widely applied in the fields of biocatalysis, biosensing, and other fields. The enzymes and their application in MOFs to immobilize POx activity are summarized in Table 2.

## 5. Application of Enzyme@MOFs in Environmental Treatment and Green Chemistry

Based on the methods of the de novo method, surface immobilization and molecular diffusion, the enzyme@MOFs composites exhibited a wider application in environmental governance and green chemistry.

In terms of environmental treatment, oxidoreductase had the ability to oxidize various pollutants that were difficult to be removed and have been widely used in the treatment of pollutants, such as dyes and heavy metals, in sewage. After immobilization, the solvent stability and tolerance of oxidoreductase were improved, and could be reused as well. In particular, MOFs, as the oxidoreductase carriers, could play a dual role in treating water pollution. In addition to serving as a carrier, MOFs compared with commonly used adsorption materials (carbonaceous materials, graphene oxides, metal oxides and metal sulphides) could adsorb heavy metal ions and dye molecules due to their extremely high porosity, high specific surface area, and adjustable functionality and structure [126,127]. Therefore, oxidoreductase@MOFs exhibit excellent application potential in environment management [128,129].

Compared to traditional chemical catalysis, biosynthesis using oxidoreductases, especially laccase and peroxidase, had the advantages of low energy consumption, moderate reaction conditions, and ecological sustainability, and is often used in textile and food industry [130]. However, the use of single oxidoreductase has certain limitations in industrial production, which often use a multi-enzyme cascade system to achieve green synthesis. Compared to traditional carriers, the unique pore size and structure of MOFs can provide sufficient space for fixing multiple enzymes so that different enzymes are in close proximity but do not interfere with each other. In addition, some MOFs themselves have catalytic ability. Therefore, oxidoreductase@MOFs composites can promote the catalytic action and improve the catalytic efficiency through multi-enzyme cascade reaction.

## 6. Conclusions and Future Work

MOFs have attracted tremendous interest as a new kind of porous material. Compared to conventional immobilization carriers, MOFs have many advantages, including high specific surface area, high porosity, diversity of species and structures, and tunable and designable organic ligands and metal nodes. Numerous studies have shown that immobilizing oxidoreductase on MOFs can increase the enzyme’s stability and recoverability as well as the range of applications for which it can be used. The composite MOFs materials fabricated by combining MOFs with organic or inorganic materials have many advantages, including electrical conductivity, mechanical stability, and better biocompatibility, which are derived from the other materials. Now, MOF-immobilized oxidoreductase has been widely developed and applied in the fields of industrial bio-sensing and -detection, biocatalysis, and recently, cancer therapy. Furthermore, MOFs are also employed in REDOX multi-enzyme cascade reactions. Here, MOFs can be used as both enzyme carriers and nano-catalysts to participate in the REDOX reactions, which shows significant application potential. 

Although the development of MOFs for oxidoreductase immobilization has made remarkable progress, there are still some challenges to be resolved. The pore channel size of MOFs is a crucial factor that affects the immobilization of oxidoreductase. Therefore, how to fabricate the MOFs with a suitable pore structure to match the enzymes’ size is the focus of future research. In addition, the interaction mechanism between enzymes and MOFs, and the effect of microenvironments composed by enzymes and MOFs or MOFs composites on catalysis activity urgently require in-depth studies, as they are helpful for the rational design and modification of MOFs. 

Furthermore, the multi-enzyme cascade systems exhibit significant potential in industrial areas due to their multifunctionalities compared to the catalytic reaction of single oxidoreductase. Consequently, the development of novel MOFs with some catalytic properties is of great interest for future study. The synergistic catalysis of MOFs catalysts and enzymes should also be investigated to meet the demands of different application fields.

## Figures and Tables

**Table 1:** Representative examples of multi-enzyme cascade reactions in MOFs.

Application	MOF	Multi-Enzyme	Refs.
Biocatalyst	HKUST-1	GOx and HRP	[78]
	ZIF-8	GOx and HRP	[80]
	Zn-MOF	GOx and HRP	[81]
	UiO-66	GOx and CPO	[84]
	UiO-66	GOx and HRP	[85]
	H-MOF(Zr)	CPO and HRP	[86]
Biosensor	ZIF-8	LAAO and HRP	[88]
	HP-DUT-5	GOx and Uricase	[89]
	ZIF-8	AChE and CHO	[90]
Bioreactor	PCN-888	GOx and HRP	[82]
	PCN-224	GOx and CAT	[87]

**Table 2:** Examples of novel MOFs in oxidoreductase-catalyzed reactions.

MOF Mimics Biological Enzymes	MOF	Enzyme	Application	Refs.
POx	Cu-MOF	GOx	Biosensor	[98]
	Cu-MOF	ChOx	Biocatalyst	[101]
	ZIF-8	GOx	Biocatalyst	[104]
	MIL-88B(Fe)	GLOX	Biosensor and Biocatalyst	[106]
	HP-PCN-222(Fe)	GOx	Biosensor	[108]
	Fe(III)-BTC	GOx	Biosensor	[109]
	NiFe2 MOF	GOx	Biocatalyst	[116]
	MnCo2O4	GOx	Biosensor	[119]
	Zr-PorMOF	GOx	Biosensor	[120]
	Co-MOF	GOx	Biosensor	[122]
	Co-TCPP(Fe)	GOx	Biosensor	[123]
	MIL-101(Fe)	GOx	Biocatalyst	[124]
	Tb-MOF	ChOx	Biosensor	[125]

## Data Availability

Not applicable.
